# Percutaneous Decannulation for Venoarterial Extracorporeal Membrane Oxygenation Using a Perclose ProGlide Closure Device and a Balloon Catheter Without On-Site Cardiac Surgical Backup

**DOI:** 10.7759/cureus.27258

**Published:** 2022-07-25

**Authors:** Tomohiro Nakamura, Shinya Murata, Ken Tsuboi, Takeshi Ishida, Shin-ichi Momomura

**Affiliations:** 1 Cardiology, Saitama Citizens Medical Center, Saitama, JPN; 2 Internal Medicine, Saitama Citizens Medical Center, Saitama, JPN

**Keywords:** percutaneous decannulation, perclose proglide, cardiogenic shock, percutaneous intervention, va-ecmo

## Abstract

Surgical decannulation for venoarterial extracorporeal membrane oxygenation (VA-ECMO) is recommended as a standard weaning strategy considering large-sized cannulas (14-22 French) are inserted in VA-ECMO. However, we should be aware of complications such as bleeding and infection when removing an arterial cannula, especially in facilities without on-site cardiac surgical backup. Percutaneous closure devices for femoral arterial access sites are currently approved for the decannulation of a 10-French or smaller sheath. We reported a case of successful weaning off from ECMO using a combination method of a balloon catheter and a Perclose ProGlide closure device. We successfully removed the arterial cannula using this technique for four ECMO-treated patients without vascular complications or blood transfusion. Percutaneous decannulation by this method could reduce the procedural time and adverse events and be safely performed even in facilities without on-site cardiac surgical backup.

## Introduction

Venoarterial extracorporeal membrane oxygenation (VA-ECMO) is feasible and effective in supporting circulation in cardiogenic shock patients [1]. The arterial and venous cannulas, which are significantly large (14-22 French) to maintain high blood flow during support, are generally introduced via the femoral vessels using the Seldinger technique. After successfully weaning off from VA-ECMO, surgical vascular repair for decannulation is considered a standard method. However, surgical closure might be associated with procedure-related complications, including bleeding, wound infection, and hemodynamic instability during the procedure [2,3]. In addition, in facilities without on-site cardiac surgical backup, manual compression hemostasis increases the risk of bleeding; therefore, complications in the removal of the VA-ECMO, especially the arterial cannula, are observed.

Currently, percutaneous closure devices of femoral arterial access sites are available for the decannulation of a 10-French or smaller sheath. The pre-close technique using the Perclose ProGlide (Abbott Vascular, Chicago, IL) closure device is now widely performed for the decannulation of larger sheaths in percutaneous endovascular aortic repair and transcatheter aortic valve replacement [4,5]. We experienced four cases of percutaneous decannulation of VA-ECMO using a combination of a balloon catheter and a Perclose ProGlide device. One case has been discussed here.

## Case presentation

A 62-year-old man collapsed after complaining of acute onset chest pain at work and was transferred by ambulance to our hospital. Despite the initiation of bystander cardiopulmonary resuscitation, the patient went into cardiac arrest with pulseless electrical activity upon arrival at the hospital. Immediately after arrival, our experienced doctors attempted to secure the bilateral femoral vessels using the Seldinger method without ultrasonography to introduce VA-ECMO in the catheterization laboratory. The arterial 15-French cannula was inserted via the right femoral artery, and a venous 21-French cannula was inserted via the left femoral vein. After the initiation of VA-ECMO support, spontaneous circulation was restored. Subsequent coronary angiography revealed three-vessel disease, and the culprit lesion of the left arterial descending artery was treated with primary stent implantation. Prior to the coronary intervention, continuous unfractionated heparin infusion (15,000 U/day) was initiated and adjusted to maintain an activated clotting time of 150-200 s, and dual antiplatelet therapies through a nasogastric tube were administered. On the second day after admission, his vital signs recovered and stabilized, and he was weaned off from VA-ECMO.

Percutaneous closure of our method

**Figure 1:**
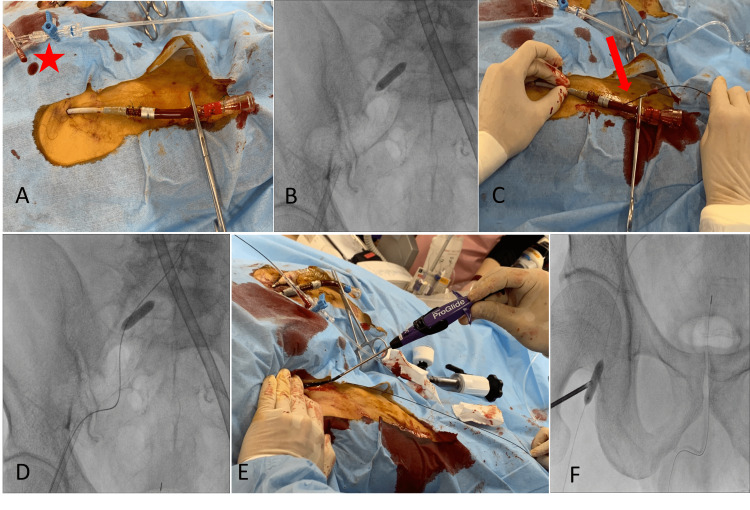
Percutaneous closure A) Clamping of the arterial cannula at the portion close to the connector. Red asterisk indicating a 6.0-mm balloon; B) a 6.0-mm balloon introduced through a 4.5-French sheath via the left radial artery was dilated in the right common iliac artery; C) the proximal portion of the arterial cannula was directly punctured using an 18-G needle, and a 0.035-inch coated guidewire (Radifocus; Terumo, Tokyo, Japan) was advanced through the arterial cannula; D) the guidewire was placed through the abdominal artery with the balloon inflated; E) after the removal of the arterial cannula, the Perclose ProGlide device was inserted along the guidewire, and suturing was performed; F) finally, the 6.0-mm balloon was re-dilated for hemostasis. If necessary, we confirmed hemostasis by angiography from a 4.5-French sheath.

We performed the removal procedure in the catheterization laboratory. The activated clotting time at the start of the procedure was 185 s. The ECMO cannulas were clamped at a portion close to the connector (Figure 1A). A 4.5-French sheath (Parent PlusTM 45; Medikit, Co., Ltd., Tokyo, Japan) was inserted into the right common iliac artery via the left radial artery, and a 6.0-mm balloon was inflated (SterlingTM; Boston Scientific, Tokyo, Japan) to reduce bleeding during the removal of the arterial cannula (Figure 1B). The proximal portion of the arterial cannula was directly punctured with an 18-G needle, and a 0.035-inch coated guidewire (Radifocus Guidewire M; Terumo, Tokyo, Japan) was inserted into the cannula (Figure 1C). The guidewire was advanced to the abdominal artery with the balloon inflated (Figure 1D). The ECMO arterial cannula was removed while the assistant manually compressed the femoral artery. The Perclose ProGlide closure device was advanced over the guidewire (Figure 1E). We performed suturing using the standard method. The 6.0-mm balloon was advanced using a 0.018-inch guidewire (Treasure Floppy®, Asahi Intecc, Co., Ltd., Aichi, Japan) to the suturing site and was reinflated for 10 min to achieve hemostasis (Figure 1F). During this process, the venous cannula was removed by manual compression for 20 min. Hemostasis could be confirmed by angiography from the sheath inserted into the iliac artery if necessary, although we did not perform it in this case. Finally, a compression bandage was applied for six hours.

In this case, the procedural time was 43 min, and complete hemostasis of both access sites was achieved. There were no vascular complications including hematoma, pseudoaneurysm, dissection, and leg ischemia after the procedure, and blood transfusion was not required during the periprocedural period.

In four ECMO-treated patients including the present case, we could successfully remove the arterial cannula using the abovementioned technique, which was a combination of a Perclose ProGlide device and a balloon. The average procedural time was approximately 50 min, and the complete hemostasis of both access sites was confirmed by angiography from the 4.5-French sheath. All patients had no vascular complications and did not require a blood transfusion during the periprocedural period.

## Discussion

We experienced a patient who successfully underwent complete percutaneous decannulation of ECMO using a combination method of a balloon catheter and a Perclose ProGlide closure device without vascular complications and blood transfusion.

Surgical closure is generally considered the standard technique for weaning off from VA-ECMO. Therefore, in facilities without on-site cardiac surgical backup, the method of decannulation of a large arterial sheath when weaning off from VA-ECMO is considered problematic. Hemostasis by manual compression has several risks, including bleeding, pseudoaneurysm, and uncertainty and extension of procedural time. The Perclose ProGlide closure device is available for hemostasis of the femoral artery access site where the large-sized sheath is inserted. A “pre”-close technique using Perclose ProGlide has been reported to be safe and effective for the closure of 21-French sheath in patients undergoing endovascular aortic aneurysm repair and transcatheter aortic valve implantation [4,5]. However, in cases of cardiogenic shock requiring ECMO, the “pre”-close technique cannot be applied due to an emergent situation, and we have to adapt the “post”-close technique. Several clinical studies showed the feasibility and safety of the percutaneous “post”-close technique using Perclose ProGlide for the decannulation of VA-ECMO [6-9]. According to the results of these previous studies, the technical success rate of the post-close technique was 86-100%, and the rate of procedure-related complications such as major bleeding requiring blood transfusion or surgical intervention, infection, leg ischemia, and pseudoaneurysm was 9-14%, which was not significantly different from surgical closure.

Our method has two unique advantages compared to previously reported methods. The first is the single post-close Perclose ProGlide technique, and the second is the combined method of percutaneous transluminal angioplasty. Previous studies on ECMO cannulation reported performing the double post-close Perclose ProGlide method, which might increase the risk of leg ischemia. Kodama et al. reported that vascular closure with a single pre-close ProGlide in transcatheter aortic valve implantation could achieve equivalent, acceptable rates of technical success, and procedural complications compared with the double pre-close ProGlide technique [10]. Even with our single post-close ProGlide method, we successfully removed the arterial cannula using the percutaneous transluminal angioplasty technique. The combined use of a balloon for angioplasty, the second unique point, allows for more reliable hemostasis. In addition to this advantage, dilating the balloon at the iliac artery could reduce bleeding during the procedure. We can confirm hemostasis at the access site by angiography from a 4.5-French sheath via the radial artery, if necessary.

Since the present report is based on only one case, it is not possible to compare the efficacy and safety of this method with those of surgical closure. We believe that it is necessary to accumulate clinical data on cases using this method and evaluate them in the future.

## Conclusions

We reported a case of a patient who was successfully weaned off from ECMO using a combination method of a balloon catheter and a Perclose ProGlide closure device. The procedural time was 43 min, and complete hemostasis could be obtained in the present case without any vascular complications or blood transfusion. Percutaneous decannulation by this method might be safely performed even in facilities without on-site cardiac surgical backup, further clinical studies are warranted.

## References

[1] Gaffney AM, Wildhirt SM, Griffin MJ, Annich GM, Radomski MW (2010). Extracorporeal life support. BMJ.

[2] Bisdas T, Beutel G, Warnecke G, Hoeper MM, Kuehn C, Haverich A, Teebken OE (2011). Vascular complications in patients undergoing femoral cannulation for extracorporeal membrane oxygenation support. Ann Thorac Surg.

[3] Aziz F, Brehm CE, El-Banyosy A, Han DC, Atnip RG, Reed AB (2014). Arterial complications in patients undergoing extracorporeal membrane oxygenation via femoral cannulation. Ann Vasc Surg.

[4] Rachel ES, Bergamini TM, Kinney EV, Jung MT, Kaebnick HW, Mitchell RA (2002). Percutaneous endovascular abdominal aortic aneurysm repair. Ann Vasc Surg.

[5] Griese DP, Reents W, Diegeler A, Kerber S, Babin-Ebell J (2013). Simple, effective and safe vascular access site closure with the double-ProGlide preclose technique in 162 patients receiving transfemoral transcatheter aortic valve implantation. Catheter Cardiovasc Interv.

[6] Majunke N, Mangner N, Linke A (2016). Comparison of percutaneous closure versus surgical femoral cutdown for decannulation of large-sized arterial and venous access sites in adults after successful weaning of veno-arterial extracorporeal membrane oxygenation. J Invasive Cardiol.

[7] Xu X, Liu Z, Han P (2019). Feasibility and safety of total percutaneous closure of femoral arterial access sites after veno-arterial extracorporeal membrane oxygenation. Medicine.

[8] Lüsebrink E, Stremmel C, Stark K (2019). Percutaneous decannulation instead of surgical removal for weaning after venoarterial extracorporeal membrane oxygenation—a crossed Perclose ProGlide Closure Device technique using a hemostasis valve Y connector. Crit Care Explor.

[9] Hwang JW, Yang JH, Sung K (2016). Percutaneous removal using Perclose ProGlide closure devices versus surgical removal for weaning after percutaneous cannulation for venoarterial extracorporeal membrane oxygenation. J Vasc Surg.

[10] Kodama A, Yamamoto M, Shimura T (2017). Comparative data of single versus double proglide vascular preclose technique after percutaneous transfemoral transcatheter aortic valve implantation from the optimized catheter valvular intervention (OCEAN-TAVI) japanese multicenter registry. Catheter Cardiovasc Interv.

